# Ultraprocessed Foods and Obesity Risk: A Critical Review of Reported Mechanisms

**DOI:** 10.1016/j.advnut.2023.04.006

**Published:** 2023-04-18

**Authors:** Vinicius M. Valicente, Ching-Hsuan Peng, Kathryn N. Pacheco, Luotao Lin, Elizabeth I. Kielb, Elina Dawoodani, Afsoun Abdollahi, Richard D. Mattes

**Affiliations:** 1 Department of Nutrition Science, Purdue University, West Lafayette, IN, United States; 2 Department of Speech, Language, and Hearing Sciences, Purdue University, West Lafayette, IN, United States; 3 Department of Human Development and Family Studies, Purdue University, West Lafayette, IN, United States

**Keywords:** ultraprocessed, NOVA, obesity, appetite, hunger, food intake, hyperpalatable, eating rate, diet

## Abstract

Epidemiologic evidence supports a positive association between ultraprocessed food (UPF) consumption and body mass index. This has led to recommendations to avoid UPFs despite very limited evidence establishing causality. Many mechanisms have been proposed, and this review critically aimed to evaluate selected possibilities for specificity, clarity, and consistency related to food choice (i.e., low cost, shelf-life, food packaging, hyperpalatability, and stimulation of hunger/suppression of fullness); food composition (i.e., macronutrients, food texture, added sugar, fat and salt, energy density, low-calorie sweeteners, and additives); and digestive processes (i.e., oral processing/eating rate, gastric emptying time, gastrointestinal transit time, and microbiome). For some purported mechanisms (e.g., fiber content, texture, gastric emptying, and intestinal transit time), data directly contrasting the effects of UPF and non-UPF intake on the indices of appetite, food intake, and adiposity are available and do not support a unique contribution of UPFs. In other instances, data are not available (e.g., microbiome and food additives) or are insufficient (e.g., packaging, food cost, shelf-life, macronutrient intake, and appetite stimulation) to judge the benefits versus the risks of UPF avoidance. There are yet other evoked mechanisms in which the preponderance of evidence indicates ingredients in UPFs actually moderate body weight (e.g., low-calorie sweetener use for weight management; beverage consumption as it dilutes energy density; and higher fat content because it reduces glycemic responses). Because avoidance of UPFs holds potential adverse effects (e.g., reduced diet quality, increased risk of food poisoning, and food wastage), it is imprudent to make recommendations regarding their role in diets before causality and plausible mechanisms have been verified.

**Statement of Significance**

This review identified no mechanistic evidence directly linking ultraprocessed food intake with increased body mass index and raises questions about adoption of the NOVA system for dietary guidance at this time.

## Introduction

It is well established that obesity is prevalent, associated with adverse health consequences, compromises the quality of life, and poses a burden on the health care system [[1], [2], [3], [4]]. Thus, developing primary and secondary prevention strategies, such as optimization of diets for individuals and the population, has been [5,6] and continues to be [7,8] a high public health priority. There is a long history of ineffective dietary recommendations aimed at preventing weight gain, so they continue to evolve. Recently, a novel perspective has gained traction. It posits that food processing is driving the obesity epidemic, rather than the nutrient composition of foods or the patterns of food intake [9]. Indeed, this has been supported by a recent review of prospective cohort studies including more than 1 million individuals [10].

To operationalize this perspective for dietary guidance, a classification system was developed, termed NOVA. It consists of 4 groups of foods, with the most problematic (i.e., most closely associated with elevated obesity risk) defined as ultraprocessed foods (UPFs) [11,12]. Definitions of UPFs have changed since it was first introduced [13], primarily to expand the problematic attributes of UPFs to include high concentrations of added sugars, salt, and fats, and to be designed expressly to be hyperpalatable, profitable, convenient, and shelf stable [14]. In addition, more recently, concerns have been raised about the presence of chemicals entering the food supply inadvertently (e.g., pesticides and acrylamide) and environmental sustainability. As a consequence, the system has moved beyond a focus on processing to one encompassing issues related to product formulation and resource management. This raises a question as to whether a unifying mechanism based on processing is now viable.

Nevertheless, acceptance of the NOVA classification system has grown to the point where moderation of UPF intake is recommended by the World Health Organization [15]; through dietary guidelines from multiple countries [1]; by scientific societies [16]; and there is sufficient concern in the United States that a review of the evidence is recommended for the 2025 Dietary Guidelines Advisory Committee (https://www/fns/usda). The topic was recently highlighted in the “Great Debates” series in the *American Journal of Clinical Nutrition* [17,18], where there was consensus by both the advocate and antagonist that epidemiologic evidence reveals an association between increasing consumption of UPFs and increasing body mass index (BMI) in the population. This has been documented in multiple systematic reviews and meta-analyses [19,20]. However, ideally, dietary recommendations are based on the convergence of epidemiologic, controlled clinical trials, and mechanistic data [1]. These 3 approaches define the scope of the problem and those at particular risk, establish causality, and characterize the mechanisms responsible. The lack of such a complete evidence base can lead to ineffective policies or ones that prove to actually increase risk and harm [[21], [22], [23]]. To date, there is insufficient documentation of a causal role played by processing because only 1, limited, randomized, controlled clinical trial has been published [24], and while multiple mechanistic options have been proposed [18], none directly link the effects of UPF intake to body weight.

Without an improved understanding of the mechanistic basis for UPF-related weight gain, the development and implementation of recommendations to alter processed food use will likely be inefficient (e.g., not target the most important elements), potentially ineffectual (e.g., low adherence due to excessive burden of unnecessary elements of behavior change), and possibly harmful (e.g., lower intake of nutrients of concern and reduced food safety). Absent knowledge of the mechanisms by which UPFs may work, potential adverse effects warrant particularly careful consideration. Many UPFs are enriched or fortified so their exclusion would run the risk of exacerbating the challenge of meeting needs for shortfall nutrients [13,25]. Indeed, data from the EPIC study revealed that highly processed foods provide between 50% and 91% of nutrients to many European countries [26]. In addition, a recent modeling study based on the Seattle Obesity Study III reported UPFs contribute most of the vitamin E, thiamin, niacin, folate, and calcium to the diet of this American cohort [26]. How or whether this would be made up with minimally processed foods is unclear. Furthermore, non–ultraprocessed foods (NUPFs) may be more expensive [27] and require more preparation time and resources than are available to segments of the population with food insecurity and very limited means. NUPFs may also have shorter shelf-lives, increasing the risk of food poisoning. A major goal of processing is to enhance the safety of the food supply. Furthermore, preservatives retard the degradation of foods, thereby protecting their nutrient quality and reducing food wastage. A recent book published by the National Academies of Sciences, Engineering and Medicine documents that ∼30% of edible food produced in the United States is wasted and much at the consumer level (∼1 pound of food per person per day) [28]. Thus, there are tangible risks associated with making dietary recommendations based on the NOVA classification system, necessitating the prior establishment of causality between UPF intake and weight gain as well as the identification of mechanisms of action.

The aim of this review was to critically evaluate evidence pertaining to multiple hypothesized mechanisms by which UPFs influence food choice, appetite, energy intake, and/or body weight/adiposity. The mechanisms to be addressed were grouped into 3 categories; those related to food choice (i.e., low cost, shelf-life, food packaging, hyperpalatability, and stimulation hunger/suppression of fullness); food composition (i.e., macronutrients, food texture, added sugar, added salt, added fat, energy density, low-calorie sweeteners (LCSs), and additives); and digestive processes (i.e., oral processing/eating rate, gastric emptying time, gastrointestinal transit time, and microbiome). The approach will be to provide a brief rationale for each hypothesized mechanism, followed by a critical assessment of the evidence relating UPF properties to ingestive behavior and indices of adiposity.

To begin, the question may be asked whether UPF intake is either necessary or sufficient for weight gain. Given that overweight/obesity in the population predates the creation of processing methods by millennia [7,29,30] and has been documented in Western nations for over 300 years [31,32], the unambiguous answer for necessity is “no." Sufficiency is also not supported because vegetarians are high consumers of UPF and yet have a low prevalence of overweight/obesity [33]. Moreover, it should be noted that although many recent meta-analyses of the relationship between consumption of UPFs and indices of body weight/adiposity document statistically significant associations [17,20,34], the odds ratios are consistently low: ranging from 1.02 [19] to 1.55 [20]. According to a recent multinational cohort study, there is only a 15% greater risk of becoming overweight or obese in people who are normal weight and a 16% greater risk of becoming obese in people who are overweight when comparing the upper and lower quintiles of UPF intake (i.e., a contrast of dietary extremes) [35]. Odds ratios are comparable or higher for numerous other drivers of obesity such as education level [36,37], economic status [37], sleep duration [38], anxiety [39], physical activity [38,40], and television viewing [38]. Thus, the level of risk associated with UPF intake does not stand out in the literature.

It is assumed in this review that the value of the NOVA classification system will lie in its ability to contribute unique information to guide healthful food choices. That is, it provides insights that do not already exist through previous studies of mechanisms driving ingestive behavior. To be sentinel, the mechanisms have to be directly linked to industrial processing practices; the purported common denominator of the classification system. That is, the association must be clear. Second, the classification system must be specific. Items in 1 food group (e.g., minimally processed) should not have the same health effects as items in another food group (e.g., ultra-processed). Lack of specificity undermines the predictability of the system. Third, the system must be internally consistent. Food properties deemed undesirable should be reliably associated with adverse weight management outcomes. If a UPF attribute actually aids weight management, rather than aggravates it, this would challenge the validity of the system. As discussed below, violations of these principles (specificity, clarity, and consistency) are more common than evidence to support them.

### Food Choice

#### Low cost

Several studies indicate that UPFs include inexpensive ingredients [41,42] so may be priced lower and are more consistently available in stores [43,44]. The cost of foods is undoubtedly an important determinant of food choice [45] and, hence, potentially, energy intake and risk for weight gain. However, whether UPFs are, by design, especially problematic is less clear. A recent systematic review and meta-analysis [46] revealed healthier choices are generally more expensive than less healthy ones, but conclusions are highly dependent on the basis of comparison (e.g., cost per calorie, cost per serving, food-based dietary pattern, and nutrient-based dietary pattern). Price differences vary substantially across foods groups, and in some cases, healthier products are comparable in cost with less-expensive options (e.g., dairy). By contrast, nutrient-based analyses that focus on daily consumption generally indicate no cost differences between more or less healthy options. The various analytical approaches not only yield discrepant outcomes but also apply to different questions. Analyses based on food patterns yield insights on price differences arising from diets that may be based on principles endorsed by the Dietary Guidelines for Americans (DGA; i.e., favoring a variety of vegetables, fruits, legumes, whole grains, low-fat or nonfat dairy, lean meats and poultry, seafood, nuts, and unsaturated vegetable oils) versus those considered highly processed. However, analyses based on food groups provide insights only into the relationship between healthfulness and price among similar foods and are less relevant for setting policy. In analyses based on dietary patterns, healthy patterns were more expensive in some instances (e.g., Mediterranean dietary pattern), but this was not true when comparisons were based on the Healthy Eating Index, Environmental Standards for Health Eating, or when comparing home-cooked to fast-food meals [46]. When standardized to 2000 kcal, these findings were reversed. This highlights that such analyses are nuanced and not simply a function of processing. Thus, the NOVA system lacks specificity on a mechanism based of cost. In addition, it is important to note that assumptions that the cost of retail goods is dictated solely by the food industry are misplaced. In reality, retail prices are determined, to a large degree, by other players in the food chain [47]. This weakens the strength of an argument that targets food industry manipulation of the price of goods to promote the intake of less healthy options and indicates limited clarity.

Regarding availability, canned and frozen items are particularly valued in households experiencing food insecurity [48] or limited (monetary or geographic) access to fresh foods because they may facilitate consumption of fruits and vegetables year-round. In particular, frozen and canned options of fruits and vegetables are generally lower in cost than their fresh counterparts [49], yet their nutritional quality is comparable. Moreover, processed fruits and vegetables have not been associated with BMI or obesity [50]. These findings indicate that processed fruits and vegetables may be cost-effective, nutritious, and accessible options to include as part of a healthy diet [49], and the argument that UPF versions of them contribute to obesity lacks consistency.

#### Shelf-life

Extended shelf-life is viewed as problematic for weight management by the NOVA system. There are both behavioral and biological considerations of this view. From a behavioral perspective, no adverse effect would be expected, but there may be benefits to consumers. For example, extending shelf-life reduces the risk of food poisoning, and the need to prepare, and likely consume portions in excess to avoid food waste (i.e., eating in the absence of hunger), which is associated with overweight/obesity [51,52]. In addition, longer product stability allows for easier planning of ingestive events, thereby reducing unplanned eating that is less likely to elicit precise energy compensation [[53], [54], [55]]. Furthermore, expanding the availability of nutrient-dense, fiber-rich, energy-dilute foods (e.g., canned, frozen and dried fruits and vegetables, canned legumes, and grain products) helps with weight management [56,57]. Thus, concern about longer shelf-life with UPFs by the NOVA system runs counter to the evidence supporting a benefit from more stable products and, therefore, lacks consistency.

Biological concerns arise from hypotheses that an expanding number of chemicals, such as selected preservatives, may function as endocrine disruptors and alter pathways related to weight management [58]. However, the extension of findings from cell culture and animal studies to a direct effect in humans is uncertain [59]. Isolating the effects of food preservatives will be difficult because endocrine disruptor chemicals are widespread in the environment and may enter the food supply inadvertently and be taken in by nondietary means (e.g., inhalation and absorption through the skin) [58,60]. Because pesticides make up the bulk of potential endocrine disruptors in food [58], exposure is unintentional. One preservative that has been identified as potentially problematic is 3-tert-butyl-4-hydroxyanisole. It reportedly phosphorylates cAMP-response element–binding protein, resulting in differentiation of preadipocytes into adipocytes in cell culture and a mouse model [61], but no data exist in humans. Another suspect class of preservatives are parabens, but again establishing their effect will be difficult because they may enter the body through inhalation, absorption through the skin, or ingestion through cosmetics and pharmaceuticals [62]. They too are hypothesized to activate adipocyte differentiation and lead to increased adiposity. However, trials in humans have yielded findings of positive [63], negative [64], and no [65] association between measures of exposure and BMI. Sodium sulfite is a generally regarded as a safe preservative added to wine (a class 1 beverage in the NOVA system). In a murine cell culture experiment, it suppressed leptin release [66]. If this were to hold in free-living humans, it could augment hunger, but there is no evidence of this action or effect in humans. Thus, at present, there is only preliminary evidence from cell culture and animal models that select preservatives hold implications for energy balance through endocrine disruption. Evidence of effects in humans is lacking and, even if true, is likely dwarfed by contributions from nonfood sources. Furthermore, these compounds are present in minimally processed foods and UPFs, diminishing their predictive value for weight outcomes. This mechanism lacks clarity, specificity, and consistency.

#### Food packaging

A feature of the NOVA classification system is that UPFs often have vivid packaging. In some instances, this is augmented by “health” claims, and in-store optimization of branded UPF, to increase purchasing and consumption of these products [12]. Multiple examples have been identified in which misplaced health connotations serve to increase purchasing, such as labeling UPF items as organic [67]. Such a claim conflates “organic” with whole ingredients and health. However, this is not specific to UPFs. A recent systematic review revealed a strong preference by consumers for products with organic labels regardless of food group (or level of processing), with environmental concerns and social responsibility as common motivators for choosing organic foods [68]. This results in low clarity for this mechanism. Other studies have demonstrated that health claims on packaging often override objective nutrition facts [69]. This is supported by a recent systematic review and meta-analysis that noted products carrying health-related claims, regardless of processing level, were more likely to be purchased and consumed [70]. These findings indicate that increased purchasing and consumption owing to health claims are not specific or clearly related to UPF but are health halos on food packaging in general.

An additional consideration for food purchasing is brand recognition and trust [71]. Many food advertisements with a strong brand recognition on food packaging are from companies that produce UPFs. Targeted and aggressive marketing has been highly effective in increasing trust and positive perceptions toward these products [71], with several studies citing in-store optimization of recognizable (UPF) brands as a facilitator of purchases, including near checkouts and end-of-aisle placement [72]. However, a recent review notes the cost of foods outweighs brand trust purchasing decisions, with negligible differences between brand name and generic alternatives of a lower price [73]. These findings indicate that although brand trust may be important to some consumers, the cost of food must be considered when making sweeping claims in the marketing of UPFs. Low cost has been cited as a feature of UPFs, but it is not a reliable indicator of the degree of processing, healthfulness, or NOVA categorization (e.g., milk, peanuts, legumes, and other foods are inexpensive but classified as group 1 foods). That is, this mechanism also lacks specificity.

#### Hyperpalatability

The sensory appeal of foods is consistently rated as the primary influence on food choice [45]. A key claim is that UPFs promote high energy intake and weight gain because they are hyperpalatable [74,75]. There is no widely agreed-on definition of hyperpalatable, but an attempt to derive one by extracting descriptions in the literature reveled it is related to the sensory qualities of fat, sugar, and salt [76]. Thus, it is largely determined by formulation rather than processing. Nevertheless, based on this analysis, 62% of the foods listed in the US Department of Agriculture Food and Nutrient Database for Dietary Studies qualify as hyperpalatable [76]. Thus, the exposure potential is high. Fat, sugar, and salt are also prevalent in UPF definitions. However, the relationship between the sensory qualities these ingredients impart, and palatability is not straightforward. Preferred dietary flavor principles for the same foods vary widely cross-culturally [[77], [78], [79]]; preferences for these sensory qualities do not grow monotonically with concentrations of fat, sugar, and salt in individuals who are lean or obese [80]; preferred sensory qualities change with age [81,82]; and hedonic ratings vary merely based on the frequency of exposure to sensory qualities [[83], [84], [85]]. These findings indicate that the criterion of hyperpalatability is not attributable to the level of processing but to biological and environmental factors. Hence, the claim about hyperpalatability lacks clarity.

It may be intuitive that highly palatable foods will be consumed in preference to less palatable options. However, because of the high palatability of the total food supply, palatability is actually a weak determinant of food choice or energy intake; accounting for <5% of the variance in energy intake [86,87]. In the only randomized controlled clinical trial contrasting the effects of minimally and UPFs, palatability did not differ between the 2 diets [24]. Energy intake did differ between the treatments, confirming palatability was not a contributor. Furthermore, another study reported the sweet taste and monosaccharide and disaccharide content of foods may be significantly associated with multiple processing categories, yet this association was actually weaker among UPFs (*r* = 0.42) than among unprocessed foods (*r* = 0.72) [88]. Thus, this mechanism lacks specificity. It should also be noted that variety has a robust effect on food intake. Preload studies of various effects on intake indicate that there is an increase in food consumption with effect sizes ranging from 15% by altering only the flavor and texture of the same food up to 40% when offering 4 different foods over successive courses [89,90]. This effect outweighs the effect of food palatability. For example, when highly palatable items such as popcorn and chocolate were offered with and without variety, the intake of both foods was significantly higher when variety was introduced [91]. Similarly, more is eaten during a meal consisting of a variety of foods than during a meal with just one of the foods, even if that food is highly palatable [92]. Finally, a systematic review noted that weight management through multiple approaches (e.g., dietary, pharmacologic, behavioral, and cognitive) generally led to reduced food reward, but this was only weakly associated with weight management [93] (i.e., weak consistency).

#### Hunger stimulate/fullness suppression

Appetitive sensations are widely believed to guide food intake. Conventional thinking holds that hunger motivates the initiation of eating events and is closely related to eating frequency, whereas fullness leads to the termination of eating events, and thus determines portion size. If either portion size or eating frequency increases without sufficient compensation by the other, energy intake will increase. With the high incidence and prevalence of overweight/obesity, it is clear that reciprocity is not strong in the current environment. Thus, there is a great interest in factors that may stimulate hunger or suppress fullness because they may be part of the problem and solution. Among these is a proposed role played by UPFs. The properties of concern include the nutritive composition and physical properties of foods. It should first be noted that appetitive influences are not as direct nor as powerful determinants of intake as often expected. A systematic review of 462 articles revealed that self-reported appetite ratings were not associated with the energy intake in 51.3% of the reports [94]. This may reflect measurement issues and the fact that decisions to eat are governed by a large number of factors with appetite being only one, and not a dominant one [95]. More often than not, people eat when they are not hungry or do not eat when they are [96] for any number of reasons (e.g., food is not available, social circumstances dictate it is or is not an appropriate time, and purposeful denial of sensations to modify body weight). Thus, this mechanism is of limited effect, generally, and prone to weak clarity and consistency.

### Food composition

#### Macronutrients

One of the most widely cited purported mechanisms involves the effect of food processing on carbohydrate metabolism. In particular, some processing practices such as the substitution of refined grains for whole grains in products may raise the glycemic index (GI) of foods. It is hypothesized that when high-GI foods are consumed, homeostatic appetitive responses are compromised. There are multiple levels of qualifications related to this phenomenon. First, the GI values of either unprocessed or processed foods are not good predictors of appetitive responses (i.e., weak clarity). Different strains of unprocessed potatoes with different GIs do not necessarily lead to differential appetitive responses (i.e., the classification lacks specificity) [97], and findings from the OmniCarb randomized clinical trial indicated that lower GI diets were associated with higher hunger [98] (a challenge to consistency). Similarly, lower hunger and greater fullness have been reported in high-GI meals [99]. Second, the NOVA system implicates added sugars and fats to foods in obesity risk. The former may increase a product’s GI value, but the latter would reduce it. Many foods with added sugars also contain a higher fat content so would not necessarily have an elevated GI value. Thus, only a subset of products with processing changes related to carbohydrate composition and form will actually result in products with higher GI values (i.e., low specificity). Third, the quantity and consumption rate of higher GI foods, the preparation method, and the composition of the other foods consumed concurrently are factors that modify the actual glycemic response (limited clarity). There is poor predictability of appetitive responses to mixed meals of low and high-GI foods [[100], [101], [102], [103], [104], [105]]. This further mitigates the predictive effects of processing. Fourth, it is assumed that high-GI foods will lead to a spike in blood glucose concentration, followed by an insulin-mediated decline, precipitating a rise in hunger. However, direct injections of glucose [106] and euglycemic clamp studies [107] demonstrate that changes in blood glucose and insulin concentrations are not causally related to hunger or fullness (poor consistency). Fifth, even if appetitive changes occur, evidence from a meta-analysis of 30 randomized controlled trials (RCTs) shows that low-GI diets do not improve body weight reduction or body fat compared with high-GI diets [108] (poor consistency). Sixth, studies that expressly tested the effects of processing on glycemic and appetitive responses fail to show an independent effect of processing [109] (low clarity). Taken together, evidence is lacking that processing-related changes in the GI value of a food or diet will translate into shifts in appetite or body weight. This conclusion is supported by another large review that included observational trials [110].

The protein leveraging hypothesis states that appetitive signaling, food intake, and body weight revolve around the consumption of dietary protein [111]. Lower protein diets are reportedly less satiating and promote a greater energy intake to meet protein needs, resulting in a higher BMI. By contrast, high-protein diets supply protein needs with less energy and should be associated with a lower BMI. Furthermore, the strong satiety effects noted with a higher protein intake are most consistently observed in solid foods [[112], [113], [114], [115], [116]]. Evidence challenging these views aside [[117], [118], [119], [120], [121], [122], [123]], the expectation would be that high-protein energy bars, which are classified as UPFs, should be beneficial for weight management. This contradicts the claim that UPF intake, as a food class, is problematic for weight management (low consistency). Furthermore, the NOVA system fails to differentiate between different protein sources that elicit discrepant effects on appetite and intake [[124], [125], [126]] (i.e., weak consistency).

Added fats are noted as problematic for weight gain in the NOVA system owing to their positive effects on palatability and weak satiety values. Views on the satiety effects of fat vary [127,128], but observations that consumption of high-fat foods leads to weak compensatory dietary responses [129,130] are evidence for its limited satiety value. One of the most marked modifications of the food supply occurred when dietary fat was viewed as a key driver of energy intake and a causal agent in the increasing incidence of overweight/obesity [131,132]. In response, the food industry marketed a large number of reduced-fat products. However, this did not abate the rising trends in BMI [133]. Thus, contrary to predictions based on the NOVA system, processing designed to improve the satiety value of foods by reducing the fat content was ineffective. Thus, this reported mechanism has weak clarity and consistency. It could be argued that the failure was due to the replacement of fat with ingredients that exacerbated the problem of positive energy balance (e.g., foods with higher GI properties). However, as discussed earlier, the greater availability of higher GI foods does not seem to be responsible. In addition, despite the acute introduction of an abundance of reduced fat foods, absolute fat intake did not decline. Indeed, it was notably stable between 1970 and ∼2011 when obesity rates continued to rise [4,[134], [135], [136], [137]]. This is inconsistent with a view that fat intake, particularly, is a primary driver of weight gain. Evidence that high-fat diets may be effective for weight management (although may pose other health risks) [[138], [139], [140], [141], [142]] or are not different from diets with higher carbohydrate or protein composition [[143], [144], [145], [146], [147]] further argue that fat poses no specific threat for weight gain. On a more subtle level, fatty acids varying in chain length and degree of saturation differ in their appetitive effects [[148], [149], [150], [151]], properties unrecognized by the NOVA system (i.e., NOVA has low specificity and consistency on this mechanism). Given that palatability is a weak predictor of energy intake, as previously discussed, the effects of added fat on energy intake due to its hedonic effect are not compelling.

Dietary fiber intake is associated with lower body weight in epidemiologic and controlled clinical studies [152]. UPFs are generally lower in fiber than minimally processed foods, so have been implicated in the problem of overweight/ obesity. The benefits of higher fiber foods, regarding energy balance, have been attributed to several mechanisms such as increased satiety through visual cues, greater orosensory processing, slower gastric emptying and gastrointestinal transit times, reduced efficiency of energy absorption, and modulation of glycemia [153]. However, these mechanistic effects are primarily demonstrable in proof-of-principle trials using extremely high levels of fiber [154]. Meta-analyses of trials examining the effects of fiber on satiety reveal very weak effects, and satiety responses were poorly correlated with actual energy intake [152]. More focused studies challenge the effect of processing itself. For example, ingestion of instant oatmeal and old-fashioned oatmeal led to comparable appetitive effects [155]. Both were superior to oat-based ready-to-eat cereal, indicating viscosity, rather than processing was the driver of the responses. These findings reveal limited clarity to this mechanism. Weak effects on appetitive sensations are consistent with findings from a meta-analysis of 26 RCTs where body weight was not reduced by wholegrain consumption compared with that by control, and only a small effect on body fat mass was observed [154] (low consistency). It may also be noted that the fiber content of several popular diets varies markedly (i.e., 4–49.1 g dietary fiber/1600 kcal) [156], but their efficacy for weight loss does not differ [143]. Thus, findings do not implicate the refining of grains in problems with weight management (effects on other health indices may be more robust). Regardless of the degree of processing, different grains and fibers may have varying effects on weight status [152,157]. The NOVA system lacks the clarity to recognize such effects.

#### Food texture

The textural properties of foods contributes to their satiation and satiety effects through the modulation of cognitive, sensory, and postingestive signaling systems [158,159]. For example, solid foods lead to higher expected [160,161] and postingestive [115] satiety than beverages; the mere addition of a nonnutritive thickener to a beverage increases its satiety value [162] and the slower gastric emptying time and gastrointestinal transit times of harder foods are associated with higher satiety [[163], [164], [165]]. UPFs are claimed to be problematic for weight gain, in part, because industrial processing disrupts the matrices of foods reducing the need for oral processing, lowering gastric emptying time, and accelerating digestion and nutrient absorption [[166], [167], [168], [169], [170], [171], [172]]. Across individuals with and without obesity, there is evidence that satiety is lower and daily energy intake is higher after consuming liquid versus solid forms of high-carbohydrate, high-protein, and high-fat foods [112] and in a trial that only altered the expectation of various physical food forms in the stomach [160]. These are inherent properties of foods, rather than processing effects (i.e., weak clarity). In addition, a recent RCT contrasting the effects of hard and soft forms of UPFs and NUPFs concluded textural properties, rather than UPF classification, were the stronger determinant of energy intake in test meals, ultimately correlating with daily energy intake [173] (i.e., low consistency).

#### Added sugar, fat, and salt

Concern about the addition of sugar, fat, and salt to foods is long-standing and widespread. All 3 were singled out in the 1977 Dietary Goals of the United States [174], the antecedents of the DGA, and remain in the current DGA. Thus, their use to define UPF in the NOVA system is redundant with current guidelines. What differs is the perspective about the roles they play. In the NOVA system, the view is that the addition of these ingredients, along with other processing practices, has led to “…the emergence of a harmful global food system and the pandemic of obesity…” [11]. UPF has also been defined as “… industrial formulations typically with 5 or more and usually many ingredients. These ingredients include items often used in processed foods, such as sugar, oils, fats, salt, antioxidants, stabilizers and preservatives.” Furthermore, proponents of the NOVA system argue that the addition of such ingredients is to “…imitate sensory qualities of group 1 foods or of culinary preparations of these foods, or to disguise undesirable sensory qualities of the final product.” Thus, their addition is viewed as unnecessary at best and possibly devious. Hence, these foods are to be avoided. By contrast, the 2020 DGA state, “The 2020-2025 Dietary Guidelines carry forward this emphasis on the importance of a healthy dietary pattern as a whole—rather than on individual nutrients, foods, or food groups in isolation.” [175]. Furthermore, they state, “…the Guidelines are a customizable framework of core elements within which individuals make tailored and affordable choices that meet their personal, cultural, and traditional preferences” [175]. That is, the DGA recognizes the importance of “preferences” and the guidance is to find a healthful dietary pattern that may include any type of food. They expressly state, “A small amount of added sugars, saturated fat, or sodium can be added to nutrient-dense foods and beverages to help meet food group recommendations…” [175]. The importance of this distinction is that simplistic and negative messaging (i.e., advising the population to avoid certain nutrients/food ingredients altogether) may have undesirable, unintended consequences [176]. Indeed, there is evidence that more liberal guidance for use of sugar [177], fat [[178], [179], [180]], and salt [[181], [182], [183]] may lead to better weight outcomes (i.e., this mechanism has low consistency).

Although the NOVA system views the addition of sugar, fat, or salt to foods negatively, there are reasons to hold a more balanced view. Sugar, fat, and salt are commonly added during food processing for purposes other than contributing to a product’s sensory properties. Sugar and salt act as humectants and inhibit microbial growth [184,185]. This reduces the risk of foodborne toxicity and permits the ingestion of nutritionally important foods year-round [186], thereby improving diet quality. The addition of fat to foods can enhance the absorption of fat-soluble nutrients [27,[187], [188], [189]]. However, additions of these ingredients generally do enhance the sensory appeal of products. This too can have beneficial nutritional effects if, by their addition, they promote the intake of nutrient-dense foods/beverages (e.g., dairy products and vegetables) in a way that meets nutrient requirements without exceeding energy needs. Consequently, the view by NOVA has weak specificity, clarity, and consistency. It should also be noted that the largest contributors of energy from fat in the US diet are fats and oils, cheese, and beef [190], and many of these foods are not categorized as “ultraprocessed” by NOVA. Foods that are highly processed, such as pretzels, cakes, cookies, and sausages, are ranked lower and do not contribute as much to total fat consumption [190]. This indicates that high-fat, energy-dense foods are not necessarily UPFs, and not all UPFs are necessarily high-fat and energy-dense (i.e., weak specificity and clarity). Furthermore, the largest contributor to the total energy consumption of individuals across the globe is staple foods such as bread, cereals, grains, poultry, pork, and fat [190]. This is particularly relevant in countries that consume home-cooked, culturally preferred foods on a regular basis; indicating that even if UPFs do increase energy intake, most of the total energy intake is derived from staple foods and not UPFs [[191], [192], [193]]. In addition, the largest sources of sodium in the diet are bread and rolls, which are not overtly salty. Moreover, although sodium intake is particularly high in Japan, the prevalence of overweight and obesity is low [194] (weak consistency).

An important question is whether the food industry adds sugar, fat, and salt to foods to stimulate intake as claimed by advocates of the NOVA system or adds them in response to consumer demand. The former assumes there is an innate liking for each of these ingredients that is exploited by the food industry to drive profits. There is strong evidence that there is an innate liking for sweet and salty qualities, with less evidence for fat [195,196]. However, there is also considerable evidence that these innate hedonic impressions are overwhelmed by environmental factors, most notably exposure frequency. This is most obviously demonstrated by cultural differences in culinary practices but has also been confirmed experimentally. The chronic addition of salt to food may lead to a preference for higher saltiness, but this is not observed with the ingestion of an equivalent amount of salt in capsules which bypasses sensory exposure [83]. Thus, meeting or exceeding salt needs does not drive intake, rather sensory experience dominates. The preferred saltiness of food can also be reduced by limiting oral salt exposure [197,198]. Similarly, restricting oral exposure to fat leads to higher acceptance of foods lower in fat, independent of fat intake [84]. Shifts in sweet preference have also been reported [85], but less consistently [199]. However, the only RCT comparing UPFs and NUPFs [200] did not observe a hedonic shift for saltiness or sweetness on either diet (low consistency), although exposures were limited to 2 weeks and the phenomenon may require 8–12 weeks to manifest. Liking is largely learned and not an inherent property of a food or its level of processing (i.e., weak clarity). It should also be noted that processing may result in products with higher salt, sugar, or fat content that is not readily perceived so is not driving intake based on their sensory qualities. There is no advantage for the food industry to drive up preferred sensation levels, by adding sugar, fat, and salt to foods because this increases the cost of foods. Current levels of additions by the food industry are just as likely to be reactionary as preemptive. Nevertheless, the current high levels of exposure do reinforce preferences, and reversing this trend will require a purposeful effort. This will require the cooperation across the food industry and their collaboration with policymakers. It is as much a formulation issue as a processing issue.

#### Energy Density

An argument implicating UPF intake with risk for overweight/obesity is that these foods generally have high energy density [18,201]. Cross-cultural analyses reveal a consistent positive association between UPF intake and dietary energy density [201,202], and it is a given that if portion sizes and eating frequency are constant, energy intake will be higher with energy-dense foods. However, observational and intervention studies reveal energy density is not significantly associated with obesity [[203], [204], [205], [206], [207], [208]]. This is not surprising given that consumption of low energy-dense foods such as fruit juices and sugar-sweetened beverages are associated with higher energy intake and body weight [[209], [210], [211], [212]] because they elicit weak energy compensation. In addition, the consumption of high energy-dense foods, such as nuts, does not lead to weight gain [[213], [214], [215], [216]] owing to a variety of mechanisms such as their high satiety value, limited energy bioavailability, and potential to increase resting energy expenditure [217]. Moreover, the primary determinants of dietary energy density, that is, water (0 kcal/g) consumption [218,219], fat (9 kcal/g) consumption [220], and fiber (1–2 kcal/g) consumption [221,222] have remained relatively stable in the American population while BMI has increased.

Although energy density is accepted as important, it is not a constant feature of UPFs. According to the NOVA classification system, the UPF group contains foods with low energy density such as soft drinks, energy/sport drinks, sweetened juices, and preprepared (packaged) vegetables [11]. Simultaneously, the unprocessed or minimally processed food group includes food items with high energy density such as nuts, seeds, grains, meat, and poultry. This results in low specificity. In the only RCT exploring the effect of the UPF diet on body weight, the diets were matched on energy density, yet body weight increased, indicating a lack of association [24]. It was argued in the study that if the beverages providing fiber in the UPF diet were excluded, energy density would be higher in the UPF diet, but there is no more physiologic basis for arbitrarily excluding the water component than any other. Water is a highly relevant dietary constituent influencing expected satiety, taste, food volume, oral processing effort and transit time, gastric emptying, and other processes linked to appetite, energy intake, and metabolism. Indeed, when water is included in the definition of energy density, associations between energy density and weight gain or BMI are reduced or not significant [223].

Although energy density (excluding beverages) was associated with weight gain in the only published RCT on UPFs [24], energy intake was declining significantly during the 2-week UPF intervention. This did not occur during the minimally processed intervention. This suggests compensatory mechanisms were active in the former, and with a longer, more nutritionally relevant time frame, the acute increase in energy intake is of questionable importance. It should also be noted that the diets in this trial supplied 81.3% and 88% of energy from UPF and NUPF, respectively, which is a far greater discrepancy than is practiced in free-living population [201,202]. The effects of more realistic levels of UPF intake have not been experimentally evaluated. So, energy density is not a clear mechanism by which UPFs contribute to obesity.

Regarding processing, technology may actually be used to reduce the energy density of foods. A good example is nanotechnology where the water and fiber content of foods may be increased [224], and the oil and fat content can be reduced [225,226] in UPFs. Such processing will decrease the energy density of UPFs and theoretically aid weight management. This results in low consistency.

#### Low-calorie sweeteners

The presence of LCSs in a product is sufficient to classify it as UPF by the NOVA system. LCSs are claimed to have a “huge negative effect” on obesity [11]. However, as documented by multiple strong meta-analyses involving prospective cohort studies and RCTs, LCSs are associated with small but statistically significant lower energy intake [[227], [228], [229], [230]] and reduced indices of adiposity (e.g., BMI, waist circumference, and body weight) [228,229,231,232]. More particularly, beverages supply 58% of added sugars and 18% of daily energy to adult diets in the United States [233], but RCTs show that consumption of LCS-sweetened beverages has a beneficial effect on weight loss and BMI compared with the consumption of sugar-sweetened beverages [[234], [235], [236], [237], [238], [239]] or possibly water [234,240]. Mechanistic studies indicate LCSs do not alter appetite [[241], [242], [243]], gut peptide secretion [244], glucose absorption [241,244], or stimulate sweetness craving [245].

There are published meta-analyses that suggest LCSs are associated with elevated BMI [246,247], but these are based on fewer studies and include trials of highly questionable relevance (e.g., analyses were limited to only individuals who gained or maintained body weight [248], administered the LCSs through capsules with effects on blood pressure as the primary outcome [[249], [250], [251]]); a sample size of 9 and trial duration of only 8 days [252], or misrepresented findings, as LCSs were associated with weight gain when the data actually indicated a slightly lower level of weight loss [250]. Thus, the evidence related to LCSs and BMI strongly supports their beneficial effects on energy balance in contrast to claims by the NOVA classification system.

Moreover, the negative view of LCSs is inconsistent with multiple goals of the NOVA system. First, added sugars are believed to contribute to weight gain. Substituting LCSs for added sugars would aid in lowering their consumption. Second, UPF’s high energy density is believed to contribute to weight gain. LCSs dilute the energy density of foods and beverages, bringing the diet more in line with the NOVA system’s goals. Third, meals high in UPF have been linked to decreased nutritional quality. Multiple studies document consumers of LCSs have higher diet quality [[253], [254], [255]]. There are multiple plausible explanations for this, one being that users are more health conscious [[256], [257], [258]] and they believe products with LCSs are healthier than their sugar-containing alternatives [[259], [260], [261], [262], [263], [264]]. Fourth, it is argued that LCSs may lead to increased energy intake because consumers overcompensate after the ingestion of reduced energy versions of foods/beverages [[265], [266], [267]]. However, this is not fully supported by clinical studies [[265], [266], [267]], and if it were true, it would apply equally to unprocessed or minimally processed foods (a problem of low specificity). A belief that a product is healthier may diminish its perceived energy content and can result in greater energy intake [268].

Taken together, LCSs are associated with beneficial effects on body weight, so to designate products containing them as UPFs runs counter to the NOVA goal of classifying foods to help consumers make choices that will aid weight management. Therefore, this mechanism has low consistency. However, preliminary evidence suggests that each LCS may evoke distinct behavioral and physiologic effects on weight gain or loss [[269], [270], [271], [272]], suggesting further focused research is warranted.

#### Additives

Hypotheses for the mechanisms underlying the high prevalence of overweight/obesity have been based largely on the energy balance equation where, for any number of biological or environmental reasons, energy intake exceeds energy expenditure, leading to positive energy balance and weight gain. However, the validity of the “energy intake-energy expenditure model” has been questioned recently based on observations that energy intake and energy expenditure have been relatively stable in the population for the past 20 y, yet the prevalence of obesity increased by over 25% [273]. Consequently, novel explanations that focus more on alterations of fuel partitioning and metabolic substrate utilization have been proposed. NOVA is, in part, a reflection of this because it posits processing effects on food matrices that alter nutrient bioaccessibility, absorption kinetics, metabolism, and the microbiome. A role played by endocrine disruptors has also been suggested [58,274] and recently included as a plausible mechanism by NOVA [275]. However, if and how purposeful food additives (e.g., colorants, preservatives, antioxidants, sweeteners, emulsifiers, stabilizers, thickeners, and gelling agents), processing products (e.g., acrylamide), and unintentional contaminants (e.g., bisphenol A, and pesticides) mechanistically affect metabolic homeostasis is poorly characterized [60]. For each claim, there is a counterclaim. For example, some suggest selected emulsifiers modify the intestinal microbiota, resulting in a shift toward proinflammatory microbial communities, which trigger a chronic inflammatory state [[276], [277], [278], [279], [280]]. Alternatively, other work indicates emulsifiers have prebiotic effects that improve and prevent gut dysbiosis and metabolic disorders [276,281]. Mixed effects of flavor compounds are also reported. The role of LCSs was described as earlier. In animal models, monosodium glutamate, a savory flavor additive, has been reported to alter energy metabolism through central nervous system pathways [282] and altered GLP-1 secretion [283], resulting in augmented weight gain. Conversely, other studies indicate monosodium glutamate intake is associated with decreased weight gain and lower body fat mass [284]. Similarly, colorants have been associated with overconsumption and weight gain [275,285,286]. However, a recent RCT indicated colored food items were associated with a lower propensity to eat compared with the food items in their original color [287]. For other proposed problematic compounds that are present in foods, there is no clear link to UPFs. Moreover, minimally processed foods, such as plant-based foods, may contain potentially problematic compounds through acceptable thermal-processing methods such as baking and roasting (e.g., acrylamide); leaching from standard packaging (e.g., bisphenols); and pesticides from farming practices. Thus, the categorization of foods on this basis lacks specificity. Furthermore, the veracity of claims that they promote obesity is unproven. For example, positive [288] and negative [289] reports are published for acrylamide and bisphenols [290,291]. Taken together, the concept that chemicals entering the food supply intentionally or unintentionally alter metabolic processes and energy balance warrants further study, but, at present, supporting evidence in humans is lacking [17,292], and the attribution selectively to UPF is questionable. Currently, this mechanism has limited consistency.

### Digestive Processes

#### Oral processing/eating rate

Food processing is a broad term that involves a wide variety of approaches for treating raw materials, such as grinding, milling, drying, cooking, frying, deboning, crushing, roasting, fermenting, freezing, pasteurization, sterilization, interesterification, hydrolysis, hydrogenation, extrusion, and other chemical modifications [11]. Through these processes, the structure of the food is often broken down into smaller particles and softer textures [293]. This is of relevance because properties of food, such as hardness, adhesiveness, springiness, cohesiveness, and chewiness, influence oral processing [294,295].

Oral processing is multidimensional. Indices include the effort exerted to prepare an ingested item for swallowing (measured variously, e.g., number of chews and muscle activity), oral transit time (exposure time), and eating rate (kilocalories consumed per minute). There is a substantial body of literature indicating that the lower processing effort required per bite; a shorter oral transit time and a faster eating rate are associated with lower satiety and higher energy intake [24,296]. Most notably, foods with harder textures lead to slower eating rates, whereas a higher eating rate is observed with liquids or foods with softer textures [112,169,297,298]. Moreover, multiple epidemiologic [299]; cross-sectional [300,301] and randomized controlled studies [302,303] (summarized in a systematic review [304]) indicate that eating rate is negatively correlated with indices of adiposity such as BMI, total fat mass percentage, android fat mass percentage, trunk fat mass, and waist circumference.

Although relationships between oral processing and obesity risk are well substantiated, the role of UPFs is unclear. Food processing can lead to greater viscosity and hardness of foods, whereas minimally processed foods can have low viscosity or hardness. Nevertheless, the proposition for UPF is that processing disrupts food matrices and reduces oral processing effort, decreases oral transit time, and increases the eating rate [305]. In one analysis, data from 5 independent studies were combined to form a data set of a wide range of different commonly consumed meals and snacks in the United Kingdom, Singapore, Germany, Netherlands, and Switzerland [305]. A total of 330 types of foods were categorized into 3 processing levels based on the NOVA classification system. The results showed that the eating rate of UPFs was significantly higher than the other 2 categories. However, it was also reported that unprocessed foods had wide eating rate variability, and some had equivalent or higher eating rates than UPFs. So, the effects of UPFs on oral processing lack specificity.

The only existing RCT on UPF intake and body weight noted that participants' eating rate was significantly greater with UPFs (vs. NUPF) [24] and this was moderately correlated (*R* = 0.45) with overall energy intake. However, energy intake was higher with UPFs for breakfast and lunch but not dinner or snacks that were also composed of either UPFs or NUPFs (low consistency). Whether eating rates differed at each meal and for snacks was not reported. Why there may be differential effects for specific eating events is unclear, but they do not correspond to UPF intake. The authors noted in the article that the suspected mechanism was the faster eating rate associated with softer food properties in UPFs. Consistent with this view, a recent randomized, controlled crossover study [173] documented that food texture is a stronger determinant of intake than the level of food processing (or UPF categorization), reducing the clarity of the mechanism. The authors noted that oral transit time was shorter with softer and liquid foods [173]. However, the validity of this claim is uncertain because other work suggests eating speed does not alter gut peptide concentrations or appetitive sensations [306]. Thus, currently, it is not possible to determine whether higher energy intake with UPFs is due to effects on oral processing. Thus, this mechanism has low consistency. Multiple articles on this topic call for RCTs to examine this mechanism [e.g., 24,305].

#### Gastric emptying time

Disruption of the matrix of foods, particularly fiber form, by processing, is proposed to accelerate gastric emptying. If true, it is posited that this will result in reduced stimulation of gastric stretch and tension mechanoreceptors [163,307,308] and lower satiation [164,[309], [310], [311], [312]], resulting in greater energy intake and weight gain. Findings consistent with aspects of this scenario are available [313]. However, the broader body of evidence fails to support this view at multiple levels. First, the physical properties of foods are not reliable predictors of gastric emptying [314]. Moreover, different effects are observed with the multiple types of fibers present in or added to foods [315] and their interactions with other food components [316]. As a recent example, porridge made from whole grain wheat (minimally processed) has a similar gastric emptying half-time compared with porridges made using 5 variations of refined, milled wheat (UPFs) [317]. Second, gastric emptying is not tightly correlated with appetitive sensations [317]. This is not surprising given that over time, individuals with partial [318,319] or complete [320] gastrectomy have normal appetitive sensations. It is clear that other systems contribute to appetitive sensations. As an example, although whole apples have a greater gastric emptying half-time compared with apple puree or juice, puree and juice have comparable gastric emptying rates [321]. However, fullness and satiety ratings were similar between whole apple and puree, but were lower for juice, revealing a poor correlation between gastric emptying time and appetite ratings. Third, many individual states and traits also influence gastric emptying [322], such as cognitive [160,323] and sensory [324] impressions, energy and macronutrient content of eating events [[325], [326], [327], [328], [329]], age [330,331], physical activity [332], and eating frequency [333]. Their interactions at each eating event hampers the prediction of gastric emptying or appetitive response to a given food, snack, or meal. Indeed, findings counter to claims regarding UPF effects are also reported where slower gastric emptying was associated with a greater energy intake and faster gastric emptying with a better appetite control [332]. Fourth, only a weak association exists between appetitive sensations and food intake [94]. Fifth, the assumption that higher energy intake at one eating event is indicative of daily or longer-term energy intake fails to recognize the presence of compensatory behaviors in children [334] and adults [335] even to UPFs [24]. Sixth, the effects of gastric distention on appetite diminish over time with chronic stimulation [336]. These points raise questions about the specificity, clarity, and consistency of this mechanism.

It should also be noted that rapid gastric emptying has also been identified as beneficial for weight management by both enhancing and suppressing appetitive sensations. Rapid emptying speeds the delivery of nutrients to the intestine where they activate a cascade of processes that enhance satiation. Indeed, combined gastric distention and intestinal signaling leads to maximal satiation [337,338]. On the contrary, if more rapid emptying leads to lower satiation, this could help individuals with selected wasting pathologies, undergoing certain treatments (e.g., chemotherapy or radiotherapy) and with advanced age who are attempting to increase energy and nutrient intake [331].

#### Intestinal transit time

Important roles have been documented for physical and endocrine influences on intestinal transit time. Appetitive signals are generated as ingesta passes through and nutrients are absorbed from the small intestine [[339], [340], [341]]. These arise from the physical and nutrient properties of foods [307,340]. However, current evidence fails to link this to UPF ingestion. The lone published RCT [24] reported that gut hormone concentrations were largely comparable at the end of the 2-week period of eating UPFs and NUPFs [24]. However, measurements were only made in the fasting state, and the prevailing view is that cognitive [160,342,343] and nutrient [[344], [345], [346], [347], [348]] signaling arising from an eating event stimulates the release of anorectic hormones and decreases the release of orexigenic hormones, resulting in shifts in appetitive sensations. Postprandial fluxes, rather than fasting concentrations, are the primary driver [342,343]. Thus, the trial [24] was not an adequate test of UPF effects. In another study, a comparison of the effects of a snack bar classified as UPF and a mixed meal revealed no differences in intestinal transit time or hunger, fullness, or postprandial satiety 4 h after ingestion [349]. This equivalence of effects for a UPF bar indicates it had a relatively higher satiety value than the meal on an energy basis. Accumulating evidence suggests total energy content of an eating event is the dominant driver of intestinal responses, rather than specific macronutrients [341], contrary to claims that the added sugars and fat content of UPFs are especially problematic. Furthermore, high levels of macronutrient intake for a few weeks may lead to gastrointestinal adaptation and return of intestinal transit time to preintervention levels [350], thereby negating acute effects. Moreover, aging [351,352] also alters intestinal processes and responses, hampering isolation of food property effects on transit time and appetitive sensations. The evidence on this mechanism presently lacks clarity and consistency.

#### Microbiome

There is an increasing interest in the role of the microbiome in modulating appetite and energy intake [[353], [354], [355], [356]] and metabolic processes associated with obesity [357,358]. Multiple mechanisms have been proposed such as the generation of bioactive compounds that alter gut hormone secretion; stimulation of the afferent vagus and brain appetitive or reward centers; alteration of responsivity to other regulatory stimuli (e.g., modify taste, reward, and appetitive centers); and enhancement of the efficiency of dietary energy extraction. However, currently, these mechanisms remain largely speculative as does a potential-independent role played by industrial processing of the food supply. Preliminary evidence shows that weight change may occur without shifts in the microbiome [359] and changes in the microbiome may occur without affecting body weight [360,361]. A recent trial examined the microbiota in adult women consuming diets rich in minimally processed foods or UPFs [362]. Both diets were associated with positive and negative changes in selected species, but UPF consumption was positively associated with species previously linked to anti-inflammatory effects. Furthermore, no significant association was observed with the overall diversity or phyla of the microbiota, nor with BMI. There is even some evidence that UPF intake may correct dysbiosis under selected conditions [363]. Much of the attention related to dietary effects on the microbiome focus on a role played by dietary fiber, but a recent large review of 107 acute studies and 29 chronic studies did not reveal clear effects of most fiber types on appetite or energy intake, and fermentability was not a significant factor [364]. Another recent meta-analysis of 39 RCTs contrasting the effects of whole grain versus refined grain intake reported effects on appetite, but these did not translate to significant effects on energy intake [365]. The authors of these analyses emphasize the variability of research methodologies, and high inter-individual variability of responsivity to interventions precludes drawing firm conclusions. Thus, the evidence base, presently, is inadequate to support UPF intake effects on the microbiome that promote weight gain, and this mechanism lacks clarity and consistency.

## Conclusion

The NOVA system attempts to provide guidance for food choices to promote health. The intent and goal are laudable. However, as highlighted in this review, there are concerns at many levels of this approach. Unlike the DGAs, the focus of NOVA is on individual behavior rather than the population. However, it does not consider individual variability in responses to given dietary exposures. It emphasizes foods to be avoided rather than promoting the positive view that all foods may be included, albeit some in limited portions and/or frequency. It expressly ignores the nutrient profiles of foods when, in the end, the primary function of eating is to acquire an adequate array of nutrients and energy. In addition, it lacks a plan for routine, critical, systematic examination of its efficacy, practicability, and safety, in contrast to the DGA’s process. Safety is of particular concern given the restriction of UPFs could jeopardize nutrient adequacy by limiting food choices; eliminating lower-cost, nutrient-fortified foods; failing to protect against food spoilage and wasting; reducing food/nutrient availability; and potentially leading to the unbalanced nutrient content of the total diet, even if selecting from minimally processed foods. Documentation of these potential negative outcomes is lacking, but their plausibility and significance necessitate further study before expanded adoption of the NOVA system. The practicality of adopting and adhering to NOVA recommendations are also questionable because the definition of UPFs has evolved and likely will continue to do so. As originally conceived, it was a plan focused on the undesirable effects of industrial food processing. This later expanded to the inclusion of certain ingredients such as added sugars, fats, and salt. At this point, the claims about processing were stretched because the issue became more about formulation than processing, yet the claims did not evolve in step. Most recently, advocates are raising concerns regarding ingredients that unintentionally enter the food chain and sustainability, so implicating not just the food industry but also the entire supply chain and resource management. The result is that NOVA is more a random collection of foods deemed less healthy on an undefined scale than a system with any common mechanism and basis for clear guidance. At this level, it is a classification scheme in which the principles are already identified and addressed by other systems such as the DGA (albeit in a less strident manner) [17]. On this last note, this review examined multiple mechanisms purported to underlie the effects of UPFs on feeding behavior and weight. It showed that UPF intake is neither sufficient nor necessary for weight gain and current effect sizes are modest. The only RCT [24] on the topic explored several mechanisms and failed to support the effects of hyperpalatability or altered appetite and did not adequately test the effects of eating rate and altered gut hormone effects. For other mechanisms (e.g., high versus low dietary fiber or texture; gastric emptying; and intestinal transit time) where there are data directly contrasting the effects of UPFs with those of NUPFs, again, no differences were observed. In other cases, data are not available (e.g., microbiome and food additives) or insufficient (e.g., packaging, food cost, shelf-life, and appetite stimulation) to judge the benefits versus the risks of UPF avoidance. There are yet other evoked mechanisms in which the preponderance of evidence indicates ingredients targeted by NOVA as unhealthy are, in fact, consistent with the goals of the NOVA classification system to moderate body weight (e.g., LCS use although it aids weight management; beverage consumption although it dilutes energy density; higher fat content although it reduces glycemic responses). Table 1 summarizes the conclusions drawn in this review on the specificity, clarity, and consistency of purported mechanisms, linking UPF intake to ingestive behavior and indices of adiposity. Although each section of this review outlined the plausibility of the various mechanisms, none were found to have a strong scientific basis. This poses a challenge to policymakers who must use the best quality scientific data to translate complex evidence into simple and clear messages. NOVA already has a message that is intuitive, but it seems clearer than the data that support it. There are other simple, clear messages that have been promoted with a better mechanistic support, such as choosing a diet that is moderate, balanced, and varied. Indeed, balance and variety are key principles of the guidance by the Food and Agriculture Organization of the United Nations and the World Health Organization for sustainable healthy diets, and a recent scoping review noted that the NOVA system stands out for its lack of consideration of these elements [366]. Guidance to consume a diet that is moderate, balanced, and varied has not been as effective at promoting health body weight as has been hoped, but this likely is due more to a problem with implementation than veracity.

**Table 1:** Summary of evidence on purported mechanisms linking UPF intake to increased obesity risk

Food choice	Mechanistic concerns		
	Specificity	Clarity	Consistency
Low cost	X	X	X
Shelf-life	X	X	X
Food packaging	X	X	
Hyperpalatability	X	X	X
Hunger stimulation/fullness suppression		X	X
Food composition			
Macronutrients	X	X	X
Food texture		X	X
Added sugar, fat, and salt	X	X	X
Energy density	X	X	X
Low-calorie sweeteners	X		X
Additives	X		X
Digestive processes			
Oral processing/eating rate	X	X	X
Gastric emptying time	X	X	X
Gastrointestinal transit time		X	X
Microbiome		X	X

Specificity—the strength of evidence that mechanisms attributed to foods/food properties in one NOVA category are unique to that category; clarity—the strength of evidence directly linking reported UPF intake effects to processing; consistency—the strength of evidence that foods/food properties in the NOVA system reliably lead to a proposed health outcome.

## Acknowledgments

The authors’ responsibilities were as follows—RDM: designed the research; VMV, C-HP, KNP, LL, EIK, ED, AA, RDM: conducted the research; VMV, C-HP, KNP, LL, EIK, ED, AA, RDM: provided essential materials; VMV, C-HP, KNP, LL, EIK, ED, AA, RDM: critically reviewed the data; VMV, C-HP, KNP, LL, EIK, ED, AA, RDM: wrote the paper; RDM: had primary responsibility for the final content; and all authors: read and approved the final manuscript.

## Funding

The authors reported no funding received for this study.

## Author disclosures

RDM serves on Scientific Advisory Boards for Mars Foods and the Grain Food Foundation and has research support from the Almond Board of California.

## Data availability

The data described in the manuscript, code book, and analytic code will not be made available because no original data were obtained and no statistical analyses were conducted.
